# A Manual of Surgical Treatment

**Published:** 1902-09

**Authors:** 


**A Manual of Surgical Treatment.** By W. WATSON CHEYNE and F. F. BURGHARD. Part VI., Section I. Pp. 479. London: Longmans, Green and Co. 1902.

In reviewing this part we need not again refer to the general scope and merits of the work as a whole. This volume is a very important one, as it deals with subjects very frequently under the care of the surgeon, and considering that so many important matters are discussed in one volume, they are as fully considered as the space could possibly allow. The operative treatment of cancer of the tongue and throat is dealt with in a very useful and thorough way, as might be expected from the work which one of the authors has done in connection with the study of this subject. The question so largely discussed at the present time—when to operate in appendicitis—is also fully considered. We are sorry the authors still try to classify cases of appendicitis on a pathological basis for clinical indications, but we are glad to see such an attempt abandoned at times—for instance, when they write of "the treatment of the more severe cases." Who shall say what the exact condition of the appendix is in such? We would also ask, What help does it give us at the bedside to call any case a "fulminating" one, as a particular type requiring special treatment? The great practical difficulty is, to define when a case is severe enough to require operation. In "severe" cases the authors advise operation at once if seen within the first twelve hours; if seen later they advise waiting for signs of suppuration, but exactly what those signs are we are not told, and really could not be. We are not yet in a position to rely on blood counts, though we may begin to try what help they can give in diagnosis. We notice that the authors advise removal of the appendix after every severe attack, even if an abscess has formed. This has been urged by other surgeons; but they advise it for an additional reason which we do not remember to have seen advanced before. They think the scar in suppurating cases so likely to yield that the appendix should be removed and the scar excised at the same time to prevent further trouble from the appendix, and also to prevent the development of a hernia. It seems to us, however, very doubtful if the appendix needs removal after every suppurating case, for in the great majority of these cases it causes no further trouble.

In operating for intractable gastric ulcer, the authors are in favour of excising the ulcer when possible; but we are rather surprised to notice gastro-enterostomy or pyloroplasty recommended in addition to this, and cannot help thinking that too much importance is attached to possible spasmodic contraction of the pylorus.

We have been struck with the excellence of the pictures which illustrate the chapters on abdominal surgery; for we have not seen such good illustrations of abdominal operations in any other book. It seems rather curious to find prescriptions containing bismuth and carbonate of soda for gastric ulcer in this surgical work. We feel inclined to remind the authors that physicians have not yet entirely retired from the practice of abdominal medicine, though their work is passing very largely over to the surgeons. Even if surgeons are not allowed to prescribe bismuth and soda, it must be remembered that the diagnosis of all forms of abdominal disease is really of greater importance to the surgeon, for he has to take the responsibility of advising as to the desirability of operation—a responsibility far greater than that incurred in prescribing drugs, or even in washing out the stomach. We regard this volume of the manual of treatment as one of the most useful of the series.

